# Effects of Low-Moderate Load High-Velocity Resistance Training on Physical Performance of Under-20 Futsal Players

**DOI:** 10.3390/sports7030069

**Published:** 2019-03-18

**Authors:** Diogo Luís Marques, Bruno Travassos, António Carlos Sousa, Maria Helena Gil, João Nuno Ribeiro, Mário Cardoso Marques

**Affiliations:** 1 Department of Sport Sciences, University of Beira Interior, 6201-001 Covilhã, Portugal; diogoluis.sequeira@gmail.com (D.L.M.); bruno.travassos@ubi.pt (B.T.); antonio_carlossousa@hotmail.com (A.C.S.); maria.helena.gil@hotmail.com (M.H.G.); joaonunorib@gmail.com (J.N.R.); 2 Research Centre in Sports, Health and Human Development, CIDESD, 6201-001 Covilhã, Portugal

**Keywords:** strength, physical performance, maximal velocity, low-to-moderate loads, low volume, futsal

## Abstract

Resistance training (RT) is an effective methodology to improve physical performance of athletes. However, up to now, no studies have addressed the RT benefits in under-20 futsal players. The purpose of this study was to evaluate the effects of six weeks of RT with high-velocity movements, low-to-moderate loads, and low volume on physical performance of under-20 futsal players. A total of 21 players were divided into two groups: A control group (CG, *n* = 10) and a RT group (RTG, *n* = 11). The RTG performed two weekly training sessions constituted by leg-press, jumps, and sprints, along with three futsal training sessions, while the CG only performed the futsal training. Before and after the intervention, the sprint time in 0–10 m (T_10_), 10–20 m (T_10–20_), and 0–20 m (T_20_), the countermovement jump (CMJ) height, the T-Test time, the kicking ball speed (KBS), and the maximum dynamic strength in the leg-press, were assessed. In post-test, significant improvements in CMJ, T-Test, KBS, and leg-press were found for the RTG, whilst a significant decrease in T_10–20_ was evidenced in the CG. The present results suggested that RT based on high velocity movements, low-to-moderate loads, and low volume produce positive effects on physical performance of under-20 futsal players.

## 1. Introduction

Futsal is a team sport where teams of five compete on a 40 × 20 m court with 20 min halves, a 10 min half-time, and unlimited substitutions [1]. It is considered an intermittent sport that requires high-intensity short duration activities of approximately 3 s, such as sprints, changes of direction, dribbles, jumps, shots, tackling, and short periods of recovery (20–30 s) during the game [2,3,4]. As a consequence of the exposure to these actions, the requirement of concentric and eccentric muscular movements in the lower limbs is very high [5], showing that futsal is one of the most physically demanding team sports [6].

According to Barbero-Alvarez et al. [7], futsal requires that each player constantly adjust his position to perform offensive and defensive activities. Due to such requirements, a player’s strength is crucial to ensure high levels of performance throughout the game. The reduction in external and internal load during the game, both in recreational athletes [8] and female futsal players [9], the decrease in the values of knee flexor and extensor torque [10], and the decline in lower-limb muscle power at the end of a match [11] reveal the importance of developing high levels of strength in futsal players [12]. For all these reasons, resistance training (RT) has been advocated as a way to increase athletic performance, but also as a means to prevent and rehabilitate injuries [6,13]. Therefore, training programs that promote strength adaptations in futsal players should be prioritized [14].

Despite its relevance, there is a large gap in the literature regarding the evaluation of different training methods on the development of appropriate strength capabilities of players according to futsal game demands [10,15]. To our knowledge, there are only two studies that aimed to analyze the effects of RT in several physical performance variables of senior futsal players [14,16]. Paz-Franco et al. [14] observed that six weeks of RT, with loads of 75% of one-repetition maximum (1RM), performed either once a week by one group or twice by another, induced significant gains in vertical jump, sprint, and repeated sprint ability (RSA) in professional futsal players. Likewise, although with light loads (45%–60% of 1RM), Torres-Torrelo et al. [16] concluded that six weeks of isolated RT (i.e., only the full squat exercise), performed by one group produced similar or greater improvements in most physical performance parameters (e.g., sprint, vertical jump, kicking ball speed, and maximal strength), except in RSA and change of direction speed, in comparison with another group that performed RT combined with an exercise of change of direction with extra load. Generally, players performed the strength exercises (e.g., squat and leg-press) with maximal velocities in the concentric phase of the movement. In the study of Paz-Franco et al. [14], significant gains in several physical performance parameters were observed. However, the effects on maximum strength were unknown, since this parameter was not assessed in that study. In opposition, Torres-Torrelo et al. [16], revealed positive effects of performing strength exercises with maximal velocities, resulting in a significant improvement on the maximum strength in both groups. Therefore, it can be stated that movement velocity used as a main training criterion has great potential to induce beneficial strength adaptations and improve athletic performance [17,18,19].

The RT methodology adopted by Torres-Torrelo et al. [16] was based on the use of low loads and low volume, rather than high loads and moderate volume as presented by Paz-Franco et al. [14]. Although there are some studies with soccer players that clearly indicate the benefits of RT using moderate-to-high loads [20,21,22], several authors [19,23,24] report that it is also possible to obtain effective strength gains using light loads, few repetitions per set, and maximal velocities. This latter methodological procedure does not promote an excessive increase in fatigue, which inhibits the capacity to produce force per unit of time and reduces the performance in different phases of the training program. Moreover, it also decreases the risk of injury [24]. Therefore, the implementation of a RT based on high-velocity movements, low-to-moderate loads, and low volume, seems to be an effective way to improve physical performance in team sports such as soccer or futsal [16,25,26,27,28].

Despite past research achievements, the effects of high-velocity RT on physical performance of under-20 futsal players remain unknown. In this way, the aim of this study is to analyze the effects of a RT program based on maximum execution velocities, low-to-moderate loads, and low volume, combined with sprints, change of direction exercises, and jumps with and without external load on the maximum dynamic strength, vertical jump height, sprint, change of direction speed, and kicking ball speed of under-20 futsal players. The hypothesis was that the group that performed six weeks of RT would obtain a significant increase in different performance parameters compared to the group that only performed the futsal training sessions.

## 2. Materials and Methods

### 2.1. Experimental Design

A quasi-experimental design was used in the present study. The players were divided into 2 groups: A control group (CG; *n* = 10) composed of players of one team and a RT group composed of players from the other team (RTG; *n* = 11). Throughout the duration of the study, both groups performed 3 futsal training sessions and one match per week. In general, the futsal training sessions started with 15 min of general exercises for warmup, 45 min of exercises for technical and tactical aspects, and 20–30 min for strategical aspects and a game. The study was carried out in the first half of the competitive season (i.e., October–December). All players were evaluated before (pre-test) and after (post-test) the intervention. The performance tests were carried out in the same week, although on different days, places, and times, according to the availability of both teams. The battery of tests was performed in two sessions, separated by an interval of 48 h. In the first session, the battery tests order was as follows: (1) 20 m sprint time, (2) countermovement jump (CMJ) height, (3) T-Test, and (4) kicking ball speed (KBS). In the second session, an additional maximum dynamic strength test was performed in the horizontal leg-press exercise. One week prior to the initial performance tests, two familiarization sessions were conducted to teach the correct execution of the tests and to minimize technical errors.

### 2.2. Subjects

A total of 25 under-20 male futsal players, from two futsal teams, volunteered to participate in the study. From the initial sample, 3 subjects were excluded due to injury and 1 due to being absent from the training sessions. Therefore, the final sample consisted of 21 subjects (5.7 ± 2.8 years of futsal experience). Both teams performed the same amount of workload during the study and also competed in the same category. None of the subjects had experience in RT. The coaches and players from both teams were previously informed of the study’s characteristics, procedures, and objectives, and all of them agreed to its terms. For players under 18 years of age, a parental written authorization to participate in the study was obtained. All the procedures followed the recommendations of the Declaration of Helsinki [29].

### 2.3. Procedures

Before the performance tests, body mass, height (Seca Instruments, Ltd., Hamburg, Germany), and body fat via bioelectrical impedance (Omron BF306) were measured. Players’ characteristics are displayed in Table 1. A warm-up protocol was performed prior to each test, which consisted of a 5 min submaximal run, followed by 3 sprints with progressive increase in velocity, 2 sets of 5 repetitions of vertical jumps (30 s rest between sets), and dynamic stretching (high knees, butt kicks, closed and open knees, hamstring kicks, leg swing towards the opposite side and walking hip, glute, quadriceps, and hamstring stretch). During the tests, all subjects were verbally encouraged to give maximal effort.

#### 2.3.1. Sprint Performance

Sprint times were recorded in an indoor pavilion with a floating wood floor. A total of 3 repetitions of 20 m sprints were performed with a 3 min rest. Photoelectric cells (Polifemo Radio Light, Microgate, Bolzano, Italy) were placed at 0, 10, and 20 m, so that the 0–10 m (T_10_), 10–20 m (T_10–20_), and 0–20 m (T_20_) times were recorded. Prior to the start of the sprints, all the subjects performed a specific warm-up constituted of 2 repetitions of 20 m sprints with progressive increase of speed. The subjects were asked to run as fast as possible after an acoustic signal (whistle), departing from a vertical position with the support foot placed forward 1 m before the first cell. The times of the 3 sprints were recorded for all subjects. Test–retest reliability for T_10_, T_10–20_, and T_20_ was assessed by the coefficient of variation (CV), presenting values of 1.09%, 1.66%, and 0.96%, respectively. The intra-class correlation coefficients (ICC) were 0.90 (95% confidence interval, CI: 0.84–0.94) for T_10_, 0.75 (CI: 0.63–0.85) for T_10–20_, and 0.90 (CI: 0.85–0.94) for T_20_. For analysis, the best time in each partial was chosen [16].

#### 2.3.2. Countermovement Jump Height

The vertical jump height, based on the measurement of the flight time, was estimated using an infrared timing system (Optojump, Microgate, Bolzano, Italy). Each subject performed 3 vertical jumps as high as possible, with both hands placed on their hips, separated by 30 s rest [30]. The CV was 3.07% and the ICC was 0.97 (CI: 0.94–0.98). The mean value of the 3 jumps was used for analysis.

#### 2.3.3. T-Test Time

**Figure 1:**
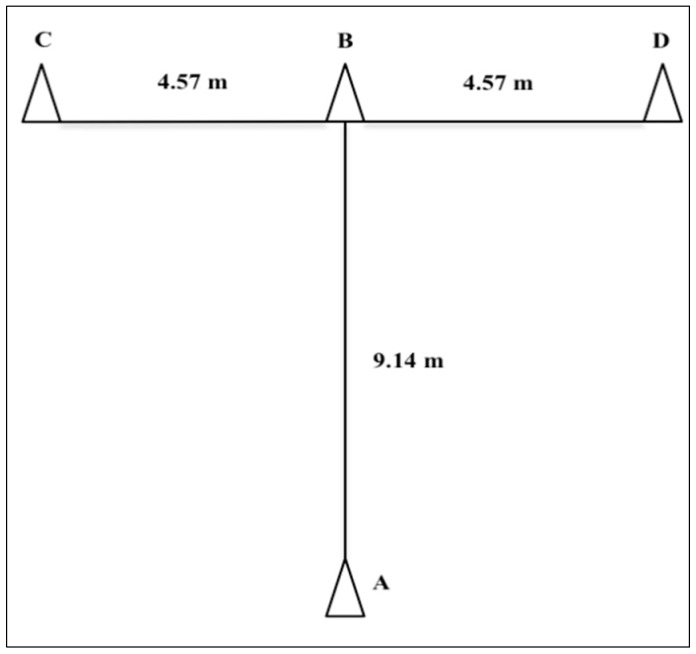
A T-Test diagram adapted from Semenick [31].

For the T-Test, 4 cones were placed in a ‘T’ shape (Figure 1) [31]. The test protocol is fully described elsewhere [32]. A total of 2 trials were performed with a 3 min rest. The times were recorded through an electronic timing gate (Brower Timing Systems, USA), placed in line with the starting point. The CV was 2.22% and the ICC was 0.75 (CI: 0.49–0.88). The best time of the 2 trials was chosen.

#### 2.3.4. Kicking Ball Speed

For the kicking ball speed, an official futsal ball Mikasa FLL555-WOR (circumference: 63.5 cm, mass: 430 g) was used. With the ball stopped 6 meters from the goal line, the subjects performed 3 kicks at maximum speed into the middle of the goal with a 1 min rest. If the ball did not reach the center of the goal, the kick was cancelled and repeated. The speed of the ball was measured using a radar gun (The Stalker Sport, Digital Sports Radar Gun, USA), placed 1 m behind the goal line between the goalposts and pointed towards the starting point of the ball. The ball speed of the 3 valid trials was recorded. The CV was 2.54% and the ICC was 0.71 (CI: 0.57–0.81). The highest ball speed of the 3 trials was used for analysis [30].

#### 2.3.5. Maximum Dynamic Strength

Maximum dynamic strength was estimated using a 1RM prediction equation for a maximal repetition test in the leg-press exercise (Leg Press G3-S70, Matrix, USA). Briefly, subjects had to sit on the bench with their head, shoulders, and back in contact with the machine, bent their knees at 90°, and placed the feet shoulder-width apart on the platform. After instruction, subjects had to fully extend their legs as fast as possible, and slowly return (3 s) to the initial position. Before the test, all subjects performed a 5 min general warm-up on a cycle-ergometer, and then a specific warm-up of 2 sets. In the first set, 5–10 repetitions at 40%–60% of the maximum load perceived were performed, followed by a 1 min rest, while in the second set, 3–5 repetitions at 60%–80% of the maximum load perceived were executed, followed by a 2 min rest. They were then instructed to perform up to a maximum of 10 repetitions with 80%–100% of the maximum load perceived. If the number of repetitions was more than 10, the load was adjusted for a second attempt. Up to 3 attempts were allowed, with a 3 min rest between them [33,34]. To estimate the value of 1RM, the predictive linear equation proposed by Brzycki [35] was used:

(1) $$1\mathrm{RM}\;=\;\frac{\mathrm{SL}}{1.0278\;-\;0.0278\;\times \;\mathrm{NR}}$$

where 1RM is the one-repetition maximum value (kg), SL is the submaximal load (kg), and NR is the number of repetitions. Predictive linear equations have been shown to be more accurate if the number of repetitions is fewer than 10 and the loads are heavy, i.e., >80% of 1RM [36,37,38]. In our study, the mean value of repetitions for the RTG in pre-test was 7 ± 2.3 and in post-test was 7 ± 2.3, while in the CG in pre-test it was 6 ± 2.3 and in post-test it was 5 ± 2.0.

### 2.4. Training Program

The training program was performed during the in-season and consisted of 2 weekly training sessions on non-consecutive days (48 h rest) for 6 weeks. In all the sessions, the subjects performed the same sequence: Sprints, change of direction (COD) exercises, depth jumps from one box to another (DJ), vertical jumps with handheld weights (VJ_W_), and the horizontal leg-press exercise. The characteristics of the training program are presented in Table 2. The training sessions took place in a gym, before the futsal training, lasting about 35 min, and were supervised by a strength and conditioning coach. All the sessions started with a 10 min warm-up (5 min of moderate intensity, followed by 2 min of submaximal intensity on a cycle ergometer, 1 set of 5 repetitions of parallel squats and vertical jumps, 2 sets of 10 m sprints and 1 min of dynamic stretching), followed by sprints of 10, 15, and 20 m, COD of 10 s, DJ of 50, 60, and 75 cm, VJ_W_ with 1 dumbbell in each hand of 2, 3, and 4 kg, and the leg-press exercise with loads of 45%–65% of 1RM. The subjects were encouraged to perform all the exercises with maximal execution velocities. Between sets and exercises, a 2–3 min rest was provided.

### 2.5. Statistical Analysis

The descriptive statistics (mean ± SD) for the different variables was calculated. Test–retest absolute reliability was assessed using the CV, while the relative reliability was assessed using the ICC, with a 95% CI. The normality and homogeneity of variance were assessed by the Shapiro–Wilk and Levene’s Test, respectively. To compare the effects of the intervention, data were analyzed using a 2 (pre- vs. post-test) × 2 (RTG vs. CG) repeated measures analysis of variance (ANOVA). If significant interaction was found, a paired-samples t-test was performed to compare the outcome variables before and after the training period. The percentage change was calculated for each variable ((Post-test – Pre-test)/Pre-test) × 100). The intra-group effect size (ES), with a CI of 90%, was also calculated using Hedge’s *g* formula [39,40]. The threshold values for assessing the magnitude of the standardized effects were 0.20, 0.60, 1.20, 2.00 for small, moderate, large, and very large, respectively [39]. Probabilities were also calculated to establish whether the true (unknown) differences were lower, similar or higher than the smallest worthwhile difference or change (0.2 × between-subject SD) [41]. Quantitative chances of better or worse effects were assessed qualitatively as follows: <1%, almost certainly not; 1%–5%, very unlikely; 5%–25%, unlikely; 25%–75%, possibly; 75%–95%, likely; 95%–99%, very likely; and >99%, almost certain. If the chances of obtaining beneficial/better or detrimental/worse were both >5%, the true difference was assessed as unclear [39,42]. Pearson’s correlation coefficients were calculated to determine associations between the percentage change in all physical performance variables. The magnitude of the correlation was assessed with the following thresholds: <0.1: Trivial; <0.1–0.3: Small; <0.3–0.5: Moderate; <0.5–0.7: Large; <0.7–0.9: Very large; <0.9–1.0: Almost perfect [39]. The statistical analysis was performed using the statistical software SPSS, version 23.0 (SPSS Inc., Chicago, IL, USA). In all the cases, the level of statistical significance was established at 0.05 (*p* < 0.05).

## 3. Results

### 3.1. Anthropometric Characteristics

In pre-test, no significant differences in the anthropometric variables were observed between both groups (Table 1). After six weeks, significant differences in the body mass variable were observed for both groups (Table 1).

### 3.2. Sprint Performance

Intra-group analysis showed that the RTG did not present significant differences in sprint performance. As for the CG, a significant decrease in T_10–20_ (*p* = 0.018) was observed. Between-group analyses showed significant differences only in T_10–20_ (*p* = 0.033) (Table 3). The RTG decreased the T_10_ (−2.05%; ES = −0.47), T_10–20_ (−0.42%; ES = −0.09), and T_20_ (−1.44%; ES = −0.36). The CG decreased the T_10_ (−0.27%; ES = −0.08) and increased the T_10–20_ (2.47%; ES = 0.77), and T_20_ (1.08%; ES = 0.35) (Table 4). The approach based on the magnitudes of change showed unclear effects for all the sprint times in the RTG, whereas the CG showed a very likely negative effect in T_10–20_ and unclear effects in T_10_ and T_20_ (Table 4).

### 3.3. Countermovement Jump Height

Intra-group comparisons revealed significant differences in the CMJ (*p* = 0.012) for the RTG. Between-group analyses revealed significant differences in the CMJ (*p* = 0.005) (Table 3). The RTG increased the CMJ height (5.60%; ES = 0.34), while the CG decreased it (−2.39%; ES = −0.27) (Table 4). The RTG showed a very likely effect in the CMJ, while the CG presented an unclear effect (Table 4).

### 3.4. T-Test Time

Intra-group comparisons showed significant improvements in the T-Test only in the RTG (*p* = 0.019). Regarding between-group interaction, there were significant differences in the T-Test (*p* = 0.008) (Table 3). The RTG decreased the T-Test time (−3.91%; ES = −0.71), while the CG increased it (1.70%; ES = 0.41) (Table 4). The magnitudes of change showed a very likely effect in the T-Test time for the RTG, whereas the CG showed an unclear effect (Table 4).

### 3.5. Kicking Ball Speed

Intra-group analysis showed significant improvements in the KBS only in the RTG (*p* = 0.004). Between-group comparisons revealed significant differences in the KBS (*p* = 0.030) (Table 3). The RTG showed an increase in the KBS (2.52%; ES = 0.437), while the CG presented a decrease (−1.02%; ES = −0.22) (Table 4). The RTG showed a most likely effect in the KBS, whereas the CG showed an unclear effect (Table 4).

### 3.6. Maximum Dynamic Strength

Intra-group analysis revealed significant improvements in the leg-press only for the RTG (*p* = 0.000). Between-group comparisons revealed significant differences in the leg-press (*p* = 0.001) (Table 3). The RTG increased the amount of weight displaced in the leg-press (17.39%; ES = 0.77), as well as the CG (1.26%; ES = 0.07) (Table 4). For the RTG, the effects in the leg-press were most likely positive, while in the CG the effects were unclear (Table 4).

### 3.7. Correlations between Changes in Physical Performance Variables

**Figure 2:**
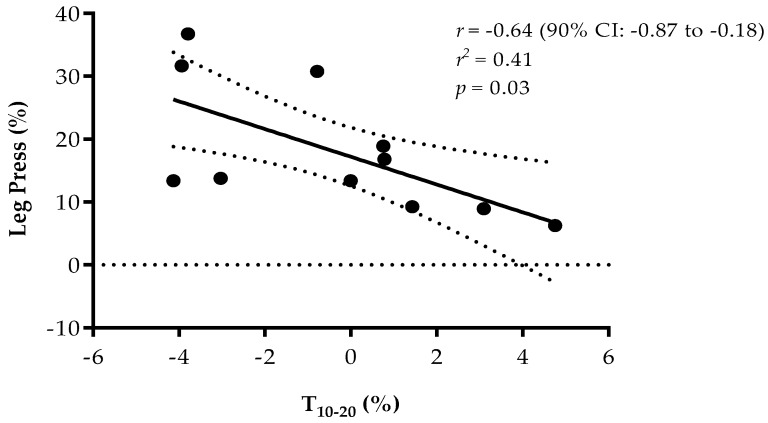
Relationship between percentage change in leg-press and percentage change in T_10–20_ performance.

In post-test, a large negative correlation between the percentage change in T_10-20_ and leg-press (*p* = 0.03; *r* = −0.64) (Figure 2), was observed. No significant correlations for the CG were observed.

## 4. Discussion

This study aimed to analyse the effects of RT with high-velocity movements, low-to-moderate loads and low volume, combined with sprints and jumps on the maximum dynamic strength, vertical jump height, sprint performance, change of direction speed and kicking ball speed in under-20 futsal players. To our knowledge, this is the first study that reports the effects of such an RT program in different strength parameters for under-20 futsal players. In general, the results support the initial hypothesis that the inclusion of an RT program combined with sprints and jumps, allows the improvement of different performance variables in under-20 futsal players during the competitive period.

### 4.1. Sprint Performance

Despite the non-significant results in all the sprint times for the RTG, both the gains (%) and the effect sizes clearly demonstrated a tendency towards an improvement on those variables. These results are in accordance with those presented in the study of Torres-Torrelo et al. [16] with senior futsal players, where the combined group (i.e., full squat + change of direction exercise) did not present significant improvements in sprint performance. However, in the study of Paz-Franco et al. [14], also with senior futsal players, the groups that performed the RT either once or twice per week presented significant improvements in sprint. These contrasting results in sprint after combined RT programs were also found in several studies with soccer players. While some studies have reported significant gains in sprint performance after combined RT [21,25,26,28,43], others did not [20,22,44,45]. According to Kobal et al. [45], there could be two factors related to the non-significant results in the sprint performance, which are the training background of the players and the short-duration of the intervention (i.e., 6 weeks). In this study, all the subjects were inexperienced in RT, which can result in greater adaptations on maximum strength and vertical jump, and less in sprint [45]. Furthermore, RT programs with longer durations seem to be most effective to induce significant gains in sprint performance, rather than RT programs with shorter durations [45]. Moreover, the in-season period might also have been a determinant factor for the non-significant improvements in sprint, since the athletes during this competitive period appear to be less sensitive to the training-induced changes [46]. Regarding the CG, a significant decrease in T_10-20_ was observed. This result was surprising, since it would be expected a maintenance or a non-significant increase in the sprint time. However, since sprinting is systematically incorporated in normal training sessions and during games, a possible induced acute fatigue could be a reasonable explanation for the reduction in sprinting performance [10].

### 4.2. Countermovement Jump Height

In this study, an improvement of 5.6% in CMJ for the RTG was observed. This result is similar to those of Torres-Torrelo et al. [16] with senior futsal players (5.4% and 6% on the combined group and the isolated RT group, respectively). Although with soccer players, gains of 5% after RT programs with light-loads and maximum execution velocities were also observed [44,47]. Thus, it seems evident that lifting light-loads with maximal velocities present, benefits in the vertical jump capacity of under-20 futsal players. Moreover, the combination of different types of jumps, with and without an external load, during the RT program may have been an important factor to improve the muscle power capacity of the subjects.

### 4.3. T-Test Time

A moderate and significant improvement was observed in T-Test in the RTG. In the study of Torres-Torrelo et al. [16], both groups presented improvements of approximately −1.7%, unlike the present study (−3.9%). However, the authors performed a different test to measure the change of direction speed (CDS), which may influence the comparison of results. In a meta-analysis performed by Silva et al. [48], the authors found that, on average, after 5–6 weeks of training, improvement of 15% in the 1RM test resulted in improvements of about −1.3% in CDS. Nevertheless, past research on CDS has presented disparate results and therefore some other factors, such as age, muscular strength level, and type of tests must be considered when interpreting the results [16,48]. The present study revealed that in order to improve CDS, loads between 45%–65% of 1RM and low volume are sufficient, instead of high-loads and high volumes, as suggested by several authors [48,49].

### 4.4. Kicking Ball Speed

A significant improvement in KBS in the RTG was observed, with an average improvement of 2.5%. This value is very similar to the results presented by Torres-Torrelo et al. [16] in both groups (~2.8%). Although those authors applied different RT methods compared to the one used in the present study, the volume and the loads were also low, and all the exercises were performed at maximum velocities. Therefore, it can be stated that the execution velocity can be a determining factor for the improvement of high-speed motor tasks. In fact, jumping exercises induce greater motor and coordinative adaptations, as well as a greater transfer of energy and strength from the proximal to the distal segments. This is largely due to the maximum velocity at which these exercises are executed, which can result in gains on kicking ability and KBS [30,50,51].

### 4.5. Maximum Dynamic Strength

A significant moderate improvement on maximum strength in the RTG was observed. The gains of 17.4% were similar to that of the isolated RT group (17%) and higher than the combined RT group (12.3%) in Torres-Torrelo et al.’s study [16] with senior futsal players. This means that a RT program with loads between 40%–65% of 1RM, low volume, and repetitions performed at maximum velocity is an effective method in improving maximum dynamic strength. In several studies with soccer players with high volume, loads between 60%–90% of 1RM, and repetitions up to or near to muscular failure, gains of 8.7% [43], 18.2% [20], 19% [52], and 22.9% [22] were observed. However, the performance improvement obtained by the RTG clearly indicates that through a light-load RT program focused on maximal execution velocities, it is possible to reach gains similar to, and even higher than the ones observed in the studies cited above.

### 4.6. Correlations between Changes in Physical Performance Variables

Regarding the large negative correlation between sprint time (T_10–20_) gains and the leg-press gains in the RTG (*r* = −0.64) (Figure 2), previous studies reported the same results after the application of RT programs [53,54,55,56,57]. Interestingly, in a study conducted by Comfort et al. [57] with 17-year-old elite soccer players, the authors found a similar strong negative correlation between maximal strength and 20 m sprint times (*r* = −0.64). In the same line and also with young elite soccer players (~18 years old), Styles et al. [52] observed that after six weeks of RT during in-season, the changes in relative 1RM squat strength correlated significantly with the changes in 5 (*r* = 0.62), 10 (*r* = 0.78), and 20 m (*r* = 0.60) sprint performances. Thus, as reported in a meta-analysis by Seitz et al. [58], increases in lower-body strength transfer positively to sprint performance, so the combined training method (resistance training along with sprints and jumps in the same session) focused on maximal execution velocities seems to be an optimal training strategy to improve sprint performance.

## 5. Conclusions

The results of the present study highlighted the fact that with using low-to-moderate loads and performing few repetitions per set with maximal execution velocities, it was possible to improve significantly different physical performance parameters of under-20 futsal players without a background in RT. In this way, our data suggested that coaches and strength and conditioning professionals should implement before futsal training, two weekly RT sessions lasting 35 min, focused on exercises with light-loads, low volume, and maximal execution velocities, in order to improve the players’ capacity to apply force as fast as possible during a rapid voluntary muscle contraction.

Nevertheless, some limitations of this study should be mentioned, such as: The sample size, the lack of studies in futsal with similar characteristics that enable the comparison of results, and finally, the non-application of a strength test based on the execution velocity that would allow us to keep the velocity as the guiding variable of the training program. Despite these limitations, the importance of performing a RT program based on low-to-moderate loads, low volume, and maximal execution velocities in under-20 futsal players was evidenced.

## Figures and Tables

**Table 1:** Anthropometric characteristics.

Group	*n*	FTE (Years)	Age (Years)	Pre-Test			Post-Test	
				Height (m)	Body Mass (kg)	Body Fat (%)	Body Mass (kg)	Body Fat (%)
RTG	11	5.2 ± 2.8	18.1 ± 0.8	1.71 ± 0.06	64.3 ± 7.0	14.8 ± 2.2	65.3 ± 7.0*	14.9 ± 2.4
CG	10	6.3 ± 2.8	17.9 ± 1.0	1.75 ± 0.12	62.5 ± 11.2	12.7 ± 2.2	64.5 ± 11.7*	13.3 ± 1.9
Total	21	5.7 ± 2.8	18.0 ± 0.9	1.73 ± 0.09	63.5 ± 9.0	13.8 ± 2.4	64.9 ± 9.3	14.1 ± 2.3

RTG: Resistance training group; CG: Control group; *n*: Number of participants; FTE: Futsal training experience; *p < 0.05 (intra-group significant differences. i.e., pre- vs. post-test).

**Table 2:** Resistance training program.

Exercises	Week 1		Week 2		Week 3		Week 4		Week 5		Week 6	
	TS1	TS2	TS3	TS4	TS5	TS6	TS7	TS8	TS9	TS10	TS11	TS12
Sprints (R × D) (m)	2 × 10 m	2 × 10 m	3 × 10 m	3 × 10 m	3 × 15 m	3 × 15 m	4 × 15 m	4 × 15 m	3 × 20 m	3 × 20 m	4 × 20 m	2 × 20 m
COD (R × T) (s)	2 × 10 s	2 × 10 s	2 × 10 s	3 × 10 s	3 × 10 s	3 × 10 s	3 × 10 s	4 × 10 s	4 × 10 s	4 × 10 s	4 × 10 s	2 × 10 s
DJ (S × R) (h)	2 × 5 × 50	2 × 5 × 50	2 × 5 × 50	2 × 5 × 50	2 × 5 × 60	2 × 5 × 60	3 × 5 × 60	3 × 5 × 60	3 × 5 × 75	3 × 5 × 75	3 × 5 × 75	1 × 5 × 75
VJ_W_ (S × R) (kg)	2 × 4 × 4	2 × 4 × 4	3 × 6 × 4	3 × 6 × 4	3 × 5 × 6	3 × 5 × 6	3 × 5 × 6	2 × 5 × 8	2 × 5 × 8	3 × 5 × 8	3 × 5 × 8	2 × 4 × 8
Leg-Press (S × R) (% 1RM)	2 × 6 × 45	2 × 6 × 45	3 × 6 × 50	3 × 6 × 50	3 × 6 × 55	3 × 6 × 55	3 × 6 × 60	3 × 6 × 60	3 × 6 × 65	3 × 6 × 65	3 × 6 × 65	2 × 5 × 60

TS: Training session; COD: Changes of direction; DJ: Depth jump from one box to another box; VJ_W_: Vertical jumps with handheld weights, i.e., one dumbbell in each hand (2 + 2 kg; 3 + 3 kg; 4 + 4 kg); R × D: Repetitions × distance; R × T: Repetitions × time; S × R: Sets × repetitions; h: Height; kg: Kilograms.

**Table 3:** Pre and post-test data (mean ± SD) and ANOVA of repeated measures results for all physical performance variables.

Variable	RTG			CG			*p* (Inter × Intra)
	Pre-Test	Post-Test	*p* (Intra)	Pre-Test	Post-Test	*p* (Intra)	
T_10_ (s)	1.86 ± 0.06	1.82 ± 0.08	0.14	1.88 ± 0.04	1.88 ± 0.08	0.79	0.29
T_10-20_ (s)	1.29 ± 0.05	1.29 ± 0.07	0.65	1.26 ± 0.04	1.29 ± 0.04	0.02*	0.03*
T_20_ (s)	3.17 ± 0.09	3.12 ± 0.14	0.15	3.15 ± 0.07	3.18 ± 0.10	0.23	0.06
CMJ (cm)	37.2 ± 5.9	39.3 ± 5.2	0.01*	37.7 ± 2.5	36.8 ± 3.5	0.20	0.00**
T-Test (s)	10.28 ± 0.41	9.88 ± 0.61	0.02*	9.69 ± 0.39	9.86 ± 0.35	0.22	0.00**
KBS (km/h)	90.8 ± 4.8	93.1 ± 4.9	0.00**	91.1 ± 2.9	90.1 ± 4.7	0.49	0.03*
LP (kg)	185.1 ± 37.4	217.3 ± 39.3	0.00***	182.9 ± 31.6	185.2 ± 32.2	0.62	0.00***

RTG: Resistance training group; CG: Control group; KBS: kicking ball speed; LP: leg-press; *p* (intra): significance value over time (pre vs. post-test); *p* (inter × intra): significance value "Group × Time"; *p < 0.05; **p < 0.01; ***p < 0.001.

**Table 4:** Percentage change from pre to post-test, effect size for within-group comparisons and intra-group magnitude-based inferences.

Variable	RTG			CG		
	Δ (90% CI)	ES (90% CI)	Percent Changes of Better/Trivial/Worse Effect	Δ (90% CI)	ES (90% CI)	Percent Changes of Better/Trivial/Worse Effect
T_10_ (s)	−2.05 (−2.92 to −1.19)	−0.48 (−1.13 to 0.18)	90/4/6 Unclear	−0.27 (−1.00 to 0.47)	−0.08 (−0.75 to 0.60)	39/36/25 Unclear
T_10-20_ (s)	−0.42 (−1.12 to 0.29)	−0.09 (−0.73 to 0.56)	32/56/16 Unclear	2.47 (1.50 to 3.44)	0.77 (0.07 to 1.46)	1/0/99 Very likely negative
T_20_ (s)	−1.44 (−2.22 to −0.65)	−0.36 (−1.01 to 0.29)	87/8/5 Unclear	1.08 (0.30 to 1.87)	0.35 (−0.33 to 1.03)	9/6/85 Unclear
CMJ (cm)	5.60 (4.05 to 7.15)	0.34 (−0.31 to 1.00)	99/1/0 Very likely positive	−2.39 (−3.35 to −1.43)	−0.27 (−0.94 to 0.41)	6/26/68 Unclear
T-Test (s)	−3.91 (−5.10 to −2.72)	−0.71 (−1.38 to −0.04)	99/1/0 Very likely positive	1.70 (0.85 to 2.56)	0.41 (−0.27 to 1.09)	8/10/82 Unclear
KBS (km/h)	2.52 (1.59 to 3.46)	0.44 (−0.22 to 1.09)	100/0/0 Most likely positive	−1.02 (−1.80 to −0.24)	−0.22 (−0.89 to 0.46)	14/36/50 Unclear
LP (kg)	17.39 (13.04 to 21.75)	0.77 (0.10 to 1.45)	100/0/0 Most likely positive	1.26 (0.46 to 2.06)	0.07 (−0.60 to 0.74)	22/66/12 Unclear

RTG: Resistance training group; CG: Control group; LP: leg-press; Δ: Percentage change; ES: Effect size Hedge´s *g*; CI: Confidence interval.
